# Supplementary material for: Reward probability and timing uncertainty alter the effect of dorsal raphe serotonin neurons on patience
Source: Nat Commun. 2018 Jun 1;9:2048. doi: 10.1038/s41467-018-04496-y (PMC5984631; doi:10.1038/s41467-018-04496-y)
Supplement: Supplementary file 1 — Supplementary Information [file 41467_2018_4496_MOESM1_ESM.pdf]

Supplementary Information

Reward probability and timing uncertainty alter the effect of dorsal raphe serotonin neurons on patience

Miyazaki et al.

Supplementaly Figures

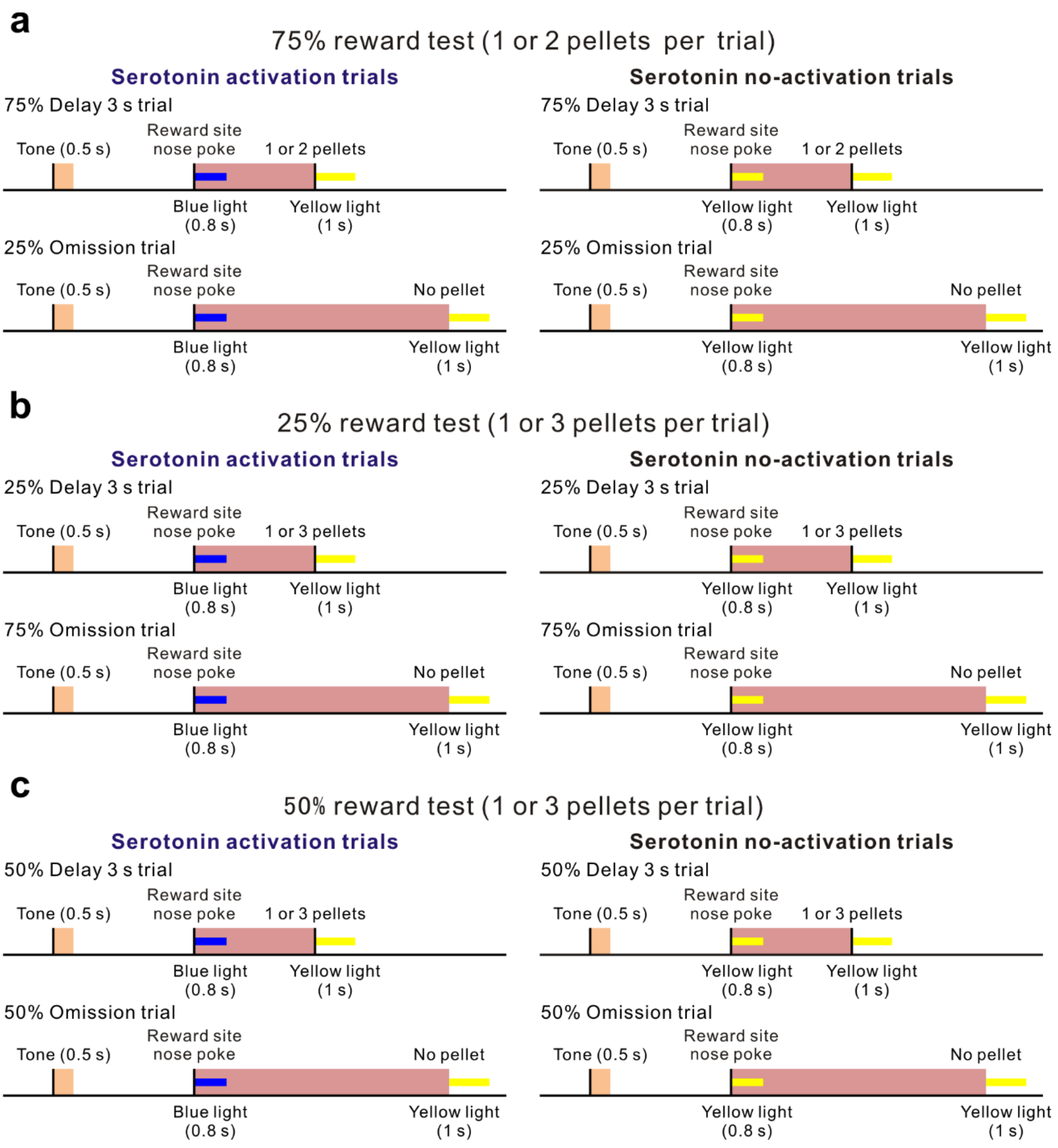

Supplementary Figure 1 | Schematic of 75%, 25% and 50% reward tests. (**a**) Time sequence of serotonin activation trials and serotonin no-activation trials in the 75% one-pellet and two pellets tests. 75% of trials were rewarded after 3 s delay. Food pellets were omitted in 25% of these trials. (**b**) Time sequence of serotonin activation and serotonin no-activation trials in the 25% one-pellet and three-pellet tests. 25% of trials were rewarded after a 3-s delay. Food pellets were omitted in 75% of these trials. (**c**) Time sequence of a serotonin activation trial and

serotonin no-activation trial in the 50% one-pellet and three-pellet tests. 50% of trials were rewarded after a 3-s delay. Food pellets were omitted in 50% of these trials. Reward and non-reward trials were randomly ordered with no sensory cues to discriminate them. In half of the trials, DRN serotonin neurons were activated by blue light (0.8 s) at the onset of the nose poke to the reward site during waiting for the delayed reward and deactivated with yellow light (1 s) upon delivery of the food pellet or the cessation of waiting. Blue and yellow bars denote blue and yellow light stimulation, respectively. Red-shaded regions denote reward delay periods. Orange-shaded regions denote duration of tone presentation.

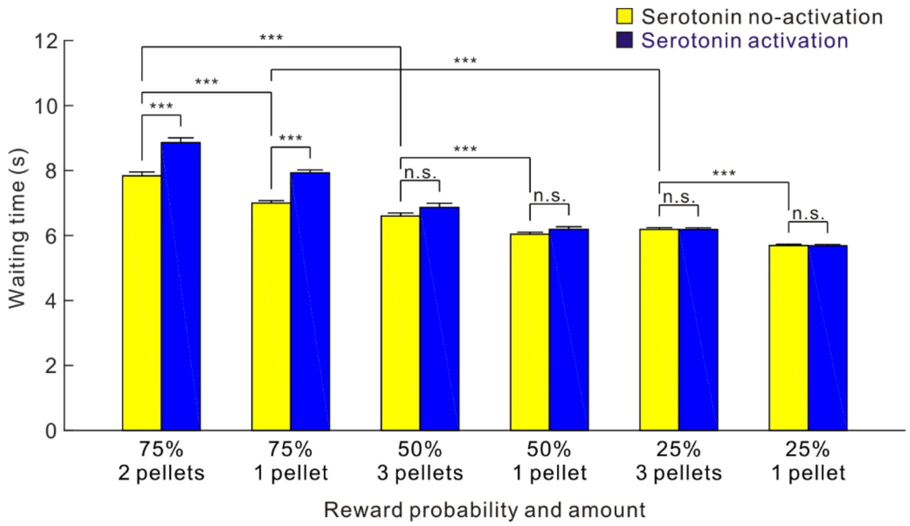

Supplementary Figure 2 | Waiting time during omission trials in the 75%, 50% and 25% reward tests. Mean waiting times of all omission trials collected from tested mice in the 75%, 50% and 25% reward tests. Optogenetic activation of serotonin neurons prolonged waiting time during omission trials in the 75% reward test, but not in 50% or 25% reward tests (75% two-pellet test, yellow, n = 145 trials from 5 mice, blue, n = 144 trials from 5 mice, U = 6732, $P = 1.80 \times 10^{-7}$; 75% one-pellet test, yellow, n = 283 trials from 6 mice, blue, n = 283 trials from 6 mice, U = 24097.5, $P = 2.45 \times 10^{-16}$; 50% three-pellet test, yellow, n = 186 trials from 3 mice, blue, n = 187 trials from 3 mice, U = 15818, P = 0.12; 50% one-pellet test, yellow, n = 210 trials from 3 mice, blue, n = 209 trials from 3 mice, U = 20390, P = 0.21; 25% three-pellet test, yellow, n = 613 trials from 6 mice, blue, n = 611 trials from 6 mice, U = 186893, P = 0.95; 25% one-pellet test, yellow, n = 569 trials from 6 mice, blue, n = 569 trials from 6 mice, U = 160568, P = 0.81). Waiting time in omission trials without serotonin activation was significantly longer in the 75%, 50% and 25% reward tests at higher reward values (75% one-pellet yellow vs. 75% two-pellet yellow, U = 11896, $P = 1.10 \times 10^{-12}$; 50% one-pellet yellow vs. 50% three-pellet yellow, U = 14274, $P = 3.78 \times 10^{-6}$; 25% one-pellet yellow vs. 25% three-pellet yellow, U = 125651.5, $P = 9.33 \times 10^{-17}$). Waiting time in omission trials without serotonin activation was significantly longer in 75% reward tests compared with tests having the same expected reward value (75% two-pellet yellow vs. 50% three-pellet yellow, U = 6047, $P = 7.29 \times 10^{-18}$; 75% one-pellet yellow vs. 25% three-pellet yellow, U = 52563.5, $P = 2.30 \times 10^{-21}$). ***$P < 0.001$ by Mann-Whitney U test. n.s., not significant. Error bars represent the s.e.m.

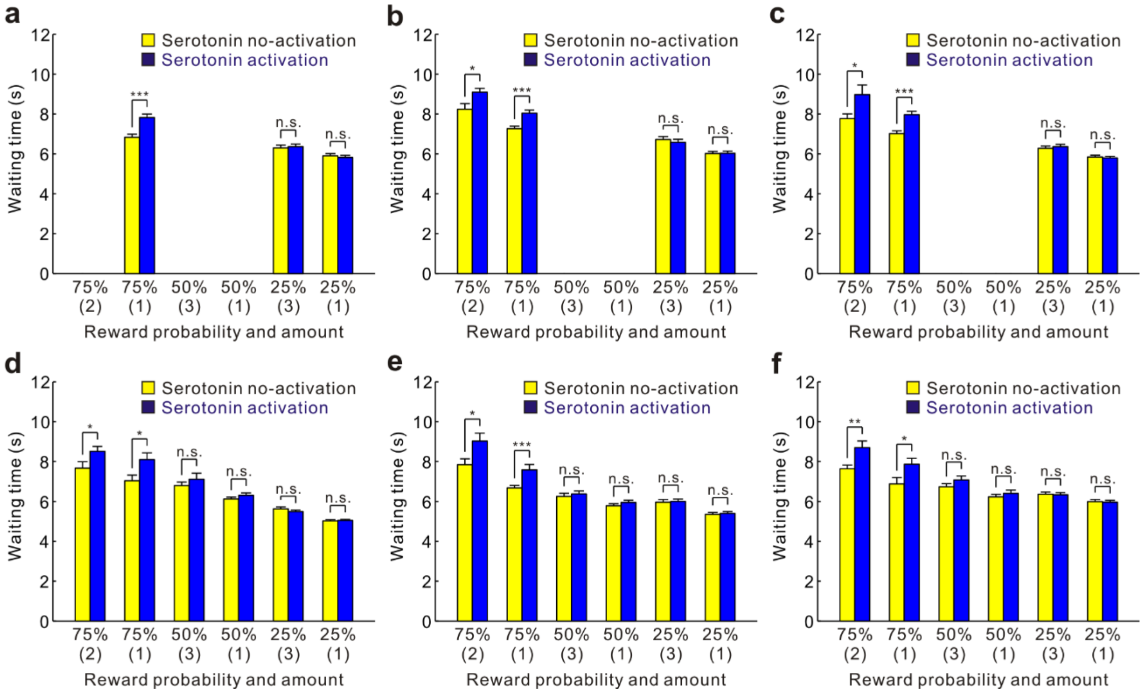

Supplementary Figure 3 | Waiting time during omission trials in Experiment 1 for each mouse. (**a-f**) Waiting time during omission trials in 75%, 50% and 25% reward tests for each of the six tested mice. (**a**) 75% one-pellet, yellow, n = 50, blue, n = 50, U = 650.5, $P = 3.63 \times 10^{-5}$; 25% three-pellet, yellow, n = 90, blue, n = 90, U = 3926, P = 0.72; 25% one-pellet, yellow, n = 75, blue, n = 75, U = 2651.5, P = 0.55. (**b**) 75% two-pellet, yellow, n = 34, blue, n = 33, U = 335, P = 0.0047; 75% one-pellet, yellow, n = 65, blue, n = 65, U = 1385, $P = 7.11 \times 10^{-4}$; 25% three-pellet, yellow, n = 104, blue, n = 103, U = 5118.5, P = 0.58; 25% one-pellet, yellow, n = 105, blue, n = 105, U = 4867, P = 0.14. (**c**) 75% two-pellet, yellow, n = 26, blue, n = 26, U = 220, P = 0.032; 75% one-pellet, yellow, n = 70, blue, n = 70, U = 1415.5, $P = 1.64 \times 10^{-5}$; 25% three-pellet, yellow, n = 120, blue, n = 120, U= 6708, P = 0.36; 25% one-pellet, yellow, n = 104, blue, n = 105, U = 5074, P = 0.38. (**d**) 75% two-pellet, yellow, n = 25, blue, n = 25, U = 190, P = 0.018; 75% one-pellet, yellow, n = 43, blue, n = 43, U = 659.5, P = 0.022; 50% three-pellet, yellow, n = 59, blue, n = 60, U = 1730, P = 0.83; 50% one-pellet, yellow, n = 70, blue, n = 70, U = 2248.5, P = 0.40, 25% three-pellet, yellow, n = 120, blue, , n = 119, U = 6506.5, P = 0.24; 25% one-pellet, yellow, n = 105, blue, n = 104, U = 5454.5, P = 0.99. (**e**) 75% two-pellet, yellow, n = 25, blue, n = 25, U = 199.5, P = 0.029; 75% one-pellet, yellow, n = 30, blue, n = 30, U = 210, $P = 3.98 \times 10^{-4}$; 50% three-pellet, yellow, n = 60, blue, n = 59, U = 1606, P = 0.35; 50% one-pellet, yellow, n = 70, blue, n = 70, U = 2246, P = 0.40, 25% three-pellet, yellow, n = 74, blue, n = 74, U = 2562, P = 0.50; 25% one-pellet, yellow, n = 75, blue, n = 75, U = 2731.5,

P = 0.76. (**f**) 75% two-pellet, yellow, n = 35, blue, n = 35, U = 402, P = 0.014; 75% one-pellet, yellow, n = 25, blue, n = 25, U = 183.5, P = 0.013; 50% three-pellet, yellow, n = 67, blue, n = 68, U = 1920, P = 0.12; 50% one-pellet, yellow, n = 70, blue, n = 69, U = 2252.5, P = 0.49, 25% three-pellet, yellow, n = 105, blue, n = 105, U = 5479.5, P = 0.94; 25% one-pellet, yellow, n = 105, blue, n = 105, U = 5231.5, P = 0.52. *P < 0.05, **P < 0.01, ***P < 0.001 by Mann-Whitney U test. n.s., not significant. Error bars represent the s.e.m.

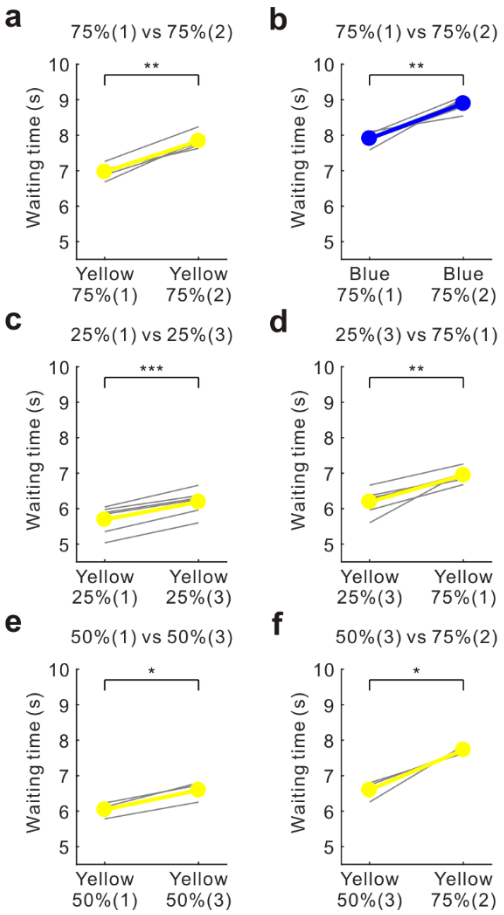

Supplementary Figure 4 | Average waiting time during omission trials depends on reward amount and reward probability. Average waiting time was significantly prolonged when reward amount was increased in the same reward probability test (**a**, **b**, **c** and **e**). Average waiting time was significantly prolonged when reward probability was increased in the same expected reward value test (**d** and **f**). (**a**) Average waiting time in serotonin no-activation during the 75% one-pellet test (yellow 75%(1)) and the 75% two-pellet test (yellow 75%(2)) for individual ChR2-expressing mice (grey lines) and for population of mice (yellow line). (**b**) Average waiting time in serotonin activation during the 75% one-pellet test (blue 75%(1)) and the 75% two-pellet test (blue 75%(2)) for individual ChR2-expressing mice (grey lines) and for population of mice (blue line). (**c**) Average waiting time in serotonin no-activation during the 25% one-pellet test (yellow 25%(1)) and the 25% three-pellet test (yellow 25%(3)) for individual ChR2-expressing mice (grey lines) and for population of mice (yellow line). (**d**) Average waiting time in serotonin no-activation during the 25% three-pellet test (yellow 25%(3)) and the 75% one-pellet test (yellow 75%(1)) for individual ChR2-expressing mice (grey lines) and for population of mice (yellow line). (**e**) Average waiting time in serotonin

no-activation during the 50% one-pellet test (yellow 50%(1)) and the 50% three-pellet test (yellow 50%(3)) for individual ChR2-expressing mice (grey lines) and for population of mice (yellow line). (**f**) Average waiting time in serotonin no-activation during the 50% three-pellet test (yellow 50%(3)) and the 75% two-pellet test (yellow 75%(1)) for individual ChR2-expressing mice (grey lines) and for population of mice (blue line). *$P < 0.05$, **$P < 0.01$, ***$P < 0.001$ by paired *t*-test. n.s., not significant. Error bars represent the s.e.m. In some case, the error bars are too small to be visible.

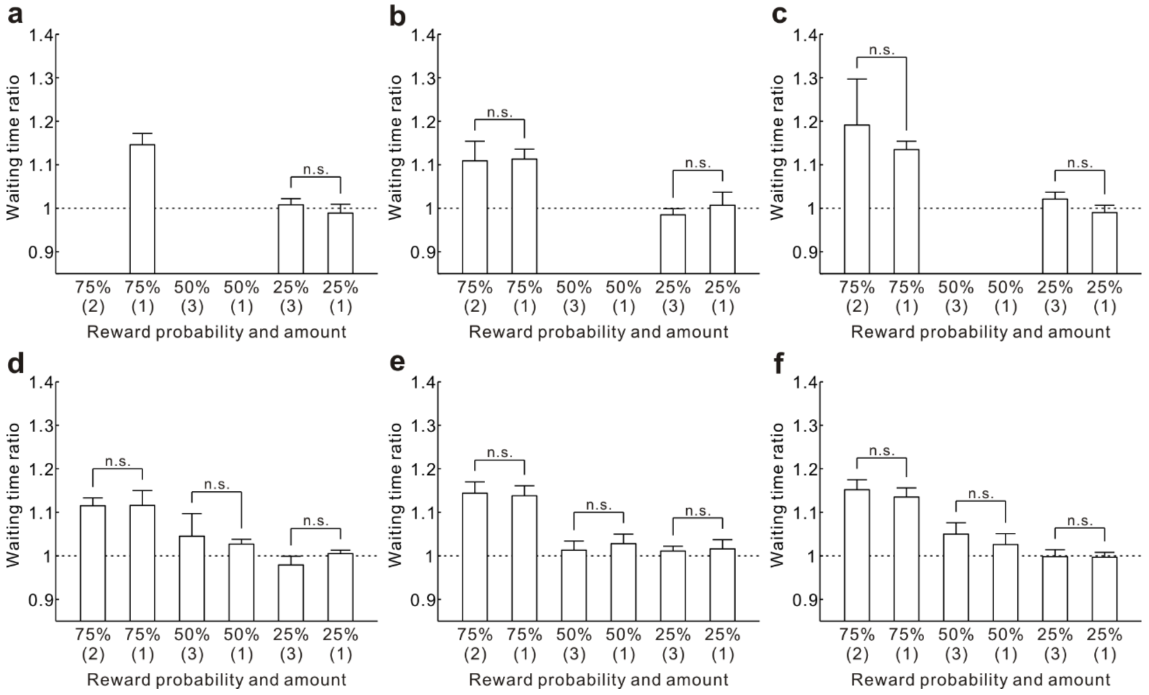

Supplementary Figure 5 | Waiting time ratio in Experiment 1 for each mouse. (**a-f**) Waiting time ratios in 75%, 50% and 25% reward tests for each of the six mice tested. (**a**) 75% one-pellet, *n* = 10; 25% three-pellet, *n* = 6; 25% one-pellet, *n* = 5; 25% three-pellet vs. 25% one-pellet, *U* = 11, *P* = 0.54. (**b**) 75% two-pellet, *n* = 7; 75% one-pellet, *n* = 13; 25% three-pellet, *n* = 7; 25% one-pellet, *n* = 7; 75% two-pellet vs. 75% one-pellet, *U* = 37.5, *P* = 0.55, 25% three-pellet vs. 25% one-pellet, *U* = 23, *P* = 0.90. (**c**) 75% two-pellet, *n* = 6; 75% one-pellet, *n* = 14; 25% three-pellet, *n* = 8; 25% one-pellet, *n* = 7; 75% two-pellet vs. 75% one-pellet, *U* = 39.5, *P* = 0.87, 25% three-pellet vs. 25% one-pellet, *U* = 16.5, *P* = 0.20. (**d**) 75% two-pellet, *n* = 5; 75% one-pellet, *n* = 9; 50% three-pellet, *n* = 6; 50% three-pellet, *n* = 7; 25% three-pellet, *n* = 8; 25% one-pellet, *n* = 7; 75% two-pellet vs. 75% one-pellet, *U* = 21, *P* = 0.90; 50% three-pellet vs. 50% one-pellet, *U* = 19, *P* = 0.84; 25% three-pellet vs. 25% one-pellet, *U* = 19, *P* = 0.34. (**e**) 75% two-pellet, *n* = 5; 75% one-pellet, *n* = 6; 50% three-pellet, *n* = 6; 50% three-pellet, *n* = 7; 25% three-pellet, *n* = 5; 25% one-pellet, *n* = 5; 75% two-pellet vs. 75% one-pellet, *U* = 13, *P* = 0.79; 50% three-pellet vs. 50% one-pellet, *U* = 16, *P* = 0.53; 25% three-pellet vs. 25% one-pellet, *U* = 12.5, *P* = 1.00. (**f**) 75% two-pellet, *n* = 7; 75% one-pellet, *n* = 5; 50% three-pellet, *n* = 7; 50% three-pellet, *n* = 7; 25% three-pellet, *n* = 7; 25% one-pellet, *n* = 7; 75% two-pellet vs. 75% one-pellet, *U* = 17, *P* = 0.96; 50% three-pellet vs. 50% one-pellet, *U* = 21, *P* = 0.69; 25% three-pellet vs. 25% one-pellet, *U* = 22, *P* = 0.80. n.s., not significant by Mann-Whitney *U* test. Error bars represent the s.e.m.

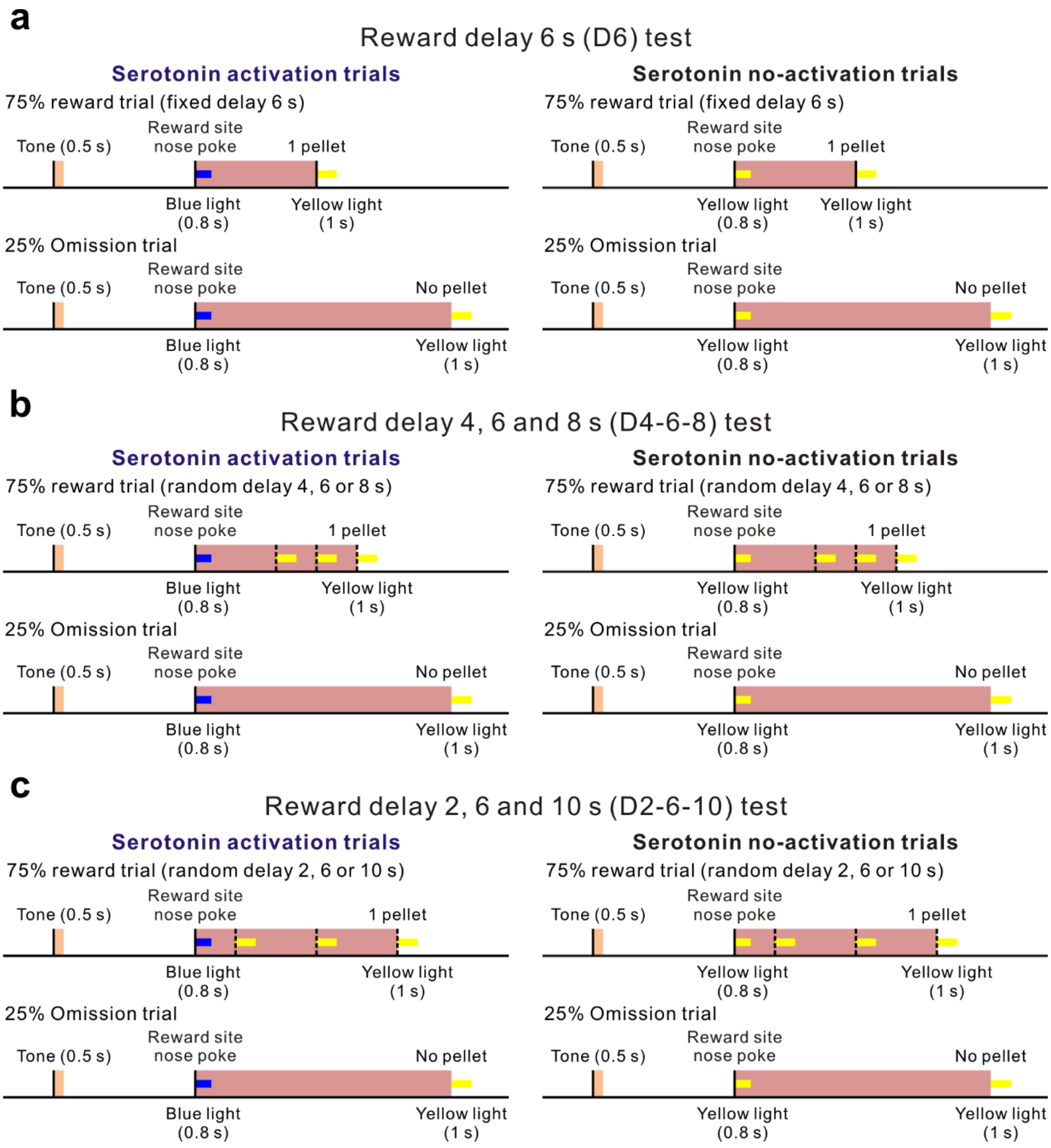

Supplementary Figure 6 | Schematic of the D6, D4-6-8 and D2-6-10 tests. (**a**) Time sequence of serotonin activation trials and serotonin no-activation trials in the D6 test. 75% of trials were rewarded after 6 s delay. Food pellets were omitted in 25% of trials. (**b**) Time sequence of serotonin activation trials and serotonin no-activation trials in the D4-6-8 test. 75% of these trials were rewarded after a 4, 6 or 8-s delay. Food pellets were omitted in 25% of trials. (**c**) Time sequence of serotonin activation trials and serotonin no-activation trials in the D2-6-10 test. 75% of these trials were rewarded after a 2, 6 or 10-s delay. Food pellets were omitted in 25% of these trials.

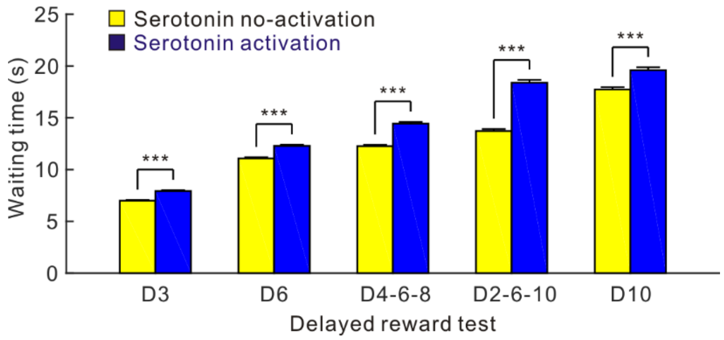

Supplementary Figure 7 | Prolongation of waiting time for future rewards with uncertain timing. Mean waiting times of all omission trials collected from tested mice in the 75% one-pellet tests in which food pellets were delivered with uncertain timing. Serotonin activation significantly prolonged waiting times for all delay conditions (D6 test, blue, n = 233 trials from 6 mice, yellow, n = 233 trials from 6 mice, U = 16945.5, $P = 2.28 \times 10^{-12}$; D4-6-8 test, blue, n = 248 trials from 6 mice, yellow, n = 246 trials from 6 mice, U = 15147.5, $P = 3.67 \times 10^{-22}$; D2-6-10 test, blue, n = 268 trials from 6 mice, yellow, n = 268 trials from 6 mice, U = 12472, $P = 4.61 \times 10^{-39}$, D10 test, blue, n = 166 trials from 4 mice, yellow, n = 166 trials from 4 mice, U = 9575.5, $P = 1.55 \times 10^{-6}$). The D3 test was the same as the 75% one-pellet test in Supplementary Figure 2. ***$P < 0.001$ by Mann-Whitney U test.

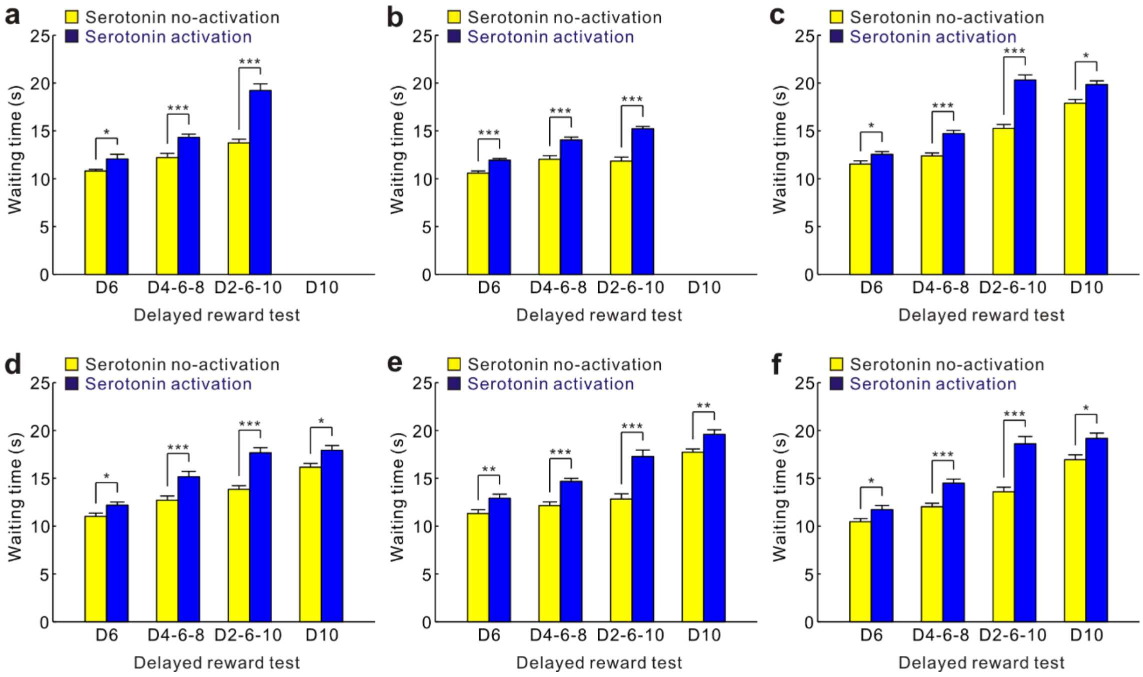

Supplementary Figure 8 | Waiting time during omission trials in Experiment 2 for each mouse. (**a-f**) Waiting time during omission trials in the D6, D4-6-8, D2-6-10 and D10 tests for each of the six tested mice. (**a**) D6 test, yellow, n = 30, blue, n = 30, U = 312.5, P = 0.043; D4-6-8 test, yellow, n = 30, blue, n = 30, U = 2323, P = 0.0013; D2-6-10 test, yellow, n = 60, blue, n = 60, U = 565.5, $P = 9.37 \times 10^{-11}$. (**b**) D6 test, yellow, n = 50, blue, n = 50, U = 613.5, $P = 1.16 \times 10^{-5}$; D4-6-8 test, yellow, n = 50, blue, n = 50, U = 563, $P = 2.22 \times 10^{-6}$; D2-6-10 test, yellow, n = 40, blue, n = 40, U = 161, $P = 8.04 \times 10^{-10}$. (**c**) D6 test, yellow, n = 63, blue, n = 64, U = 1337, P = 0.0011; D4-6-8 test, yellow, n = 60, blue, n = 60, U = 856.5, $P = 7.44 \times 10^{-7}$; D2-6-10 test, yellow, n = 59, blue, n = 58, U = 483.5, $P = 2.25 \times 10^{-11}$; D10 test, yellow, n = 42, blue, n = 44, U = 634, P = 0.012. (**d**) D6 test, yellow, n = 30, blue, n = 30, U = 286.5, P = 0.016; D4-6-8 test, yellow, n = 30, blue, n = 30, U = 234, P = 0.0014; D2-6-10 test, yellow, n = 35, blue, n = 35, U = 178.5, $P = 3.54 \times 10^{-7}$; D10 test, yellow, n = 45, blue, n = 44, U = 712.5, P = 0.023. (**e**) D6 test, yellow, n = 29, blue, n = 30, U = 284, P = 0.023; D4-6-8 test, yellow, n = 33, blue, n = 34, U = 1496.5, $P = 2.01 \times 10^{-5}$; D2-6-10 test, yellow, n = 30, blue, n = 29, U = 131, $P = 4.19 \times 10^{-6}$; D10 test, yellow, n = 39, blue, n = 38, U = 423, P = 0.0012. (**f**) D6 test, yellow, n = 30, blue, n = 30, U = 259, P = 0.0049; D4-6-8 test, yellow, n = 44, blue, n = 43, U = 515.5, $P = 2.62 \times 10^{-4}$; D2-6-10 test, yellow, n = 45, blue, n = 45, U = 280, $P = 3.39 \times 10^{-7}$; D10 test, yellow, n = 40, blue, n = 40, U = 523.5, P = 0.0079. (b) and (a) in Supplementary Figure 3, (c) and (b) in Supplementary Figure 3, (d) and (c) in Supplementary Figure 3, (e) and (e) in Supplementary Figure 3, and (f) and (f) in Supplementary Figure 3 were the same mouse,

respectively. $*P < 0.05$, $**P < 0.01$, $***P < 0.001$ by Mann-Whitney *U* test. Error bars represent the s.e.m.

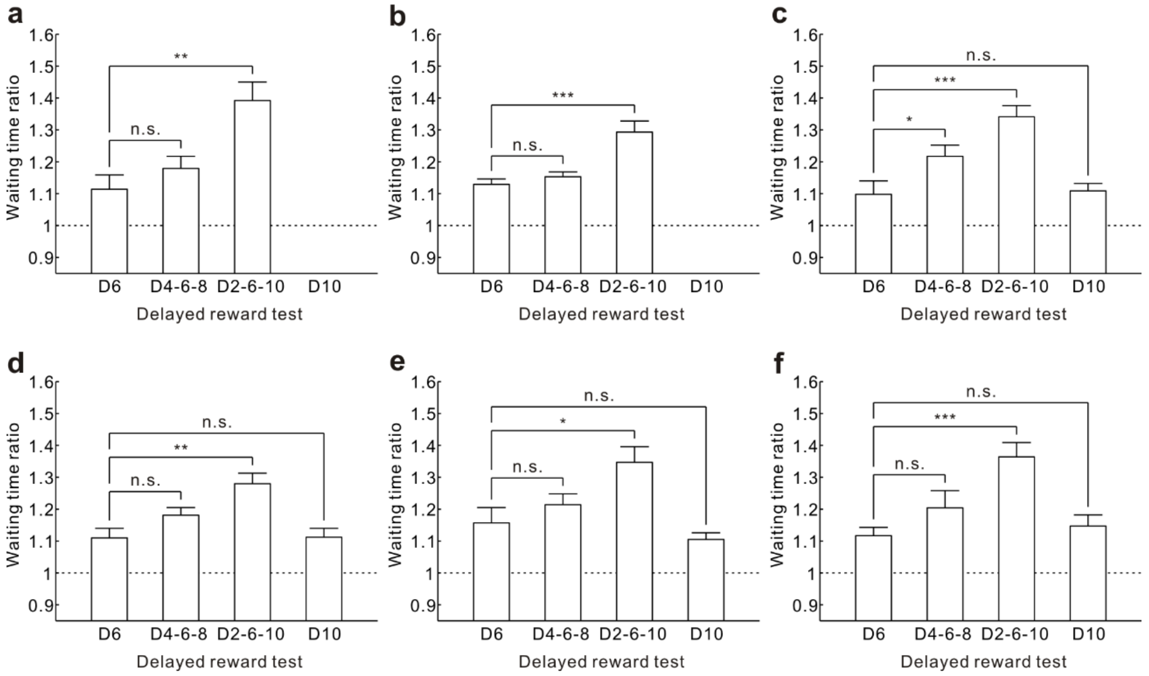

Supplementary Figure 9 | Waiting time ratio in Experiment 2 for each mouse. (**a-f**) Waiting time ratios in the D6, D4-6-8, D2-6-10 and D10 tests for each of the six tested mice. (**a**) D6 test, n = 6; D4-6-8 test, n = 6; D2-6-10 test, n = 12; D6 vs. D4-6-8, U = 8.5, P = 0.15; D6 vs. D2-6-10, U = 6, P = 0.0032. (**b**) D6 test, n = 10; D4-6-8 test, n = 10; D2-6-10 test, n = 8; D6 vs. D4-6-8, U = 43.5, P = 0.65; D6 vs. D2-6-10, U = 27, $P = 1.83 \times 10^{-4}$. (**c**) D6 test, n = 13; D4-6-8 test, n = 12; D2-6-10 test, n = 12; D10 test, n = 9; D6 vs. D4-6-8, U = 34, P = 0.018; D6 vs. D2-6-10, U = 1, $P = 3.15 \times 10^{-5}$. D6 vs. D10, U= 57, P = 0.95. (**d**) D6 test, n = 6; D4-6-8 test, n = 6; D2-6-10 test, n = 7; D10 test, n = 9; D6 vs. D4-6-8, U = 7, P = 0.089; D6 vs. D2-6-10, U = 1, P = 0.0023. D6 vs. D10, U= 27, P = 1. (**e**) D6 test, n = 6; D4-6-8 test, n = 7; D2-6-10 test, n = 6; D10 test, n = 8; D6 vs. D4-6-8, U = 11, P = 0.18; D6 vs. D2-6-10, U = 3, P = 0.015; D6 vs. D10, U= 16.5, P = 0.36. (**f**) D6 test, n = 6; D4-6-8 test, n = 9; D2-6-10 test, n = 9; D10 test, n = 8; D6 vs. D4-6-8, U = 15.5, P = 0.19; D6 vs. D2-6-10, U = 1, $P = 7.99 \times 10^{-4}$. D6 vs. D10, U = 22.5, P = 0.88. *P < 0.05, **P < 0.01, ***P < 0.001 by Mann-Whitney U test. n.s., not significant. Error bars represent the s.e.m.

Supplementary Tables

Supplementary Table 1 | Difference of means between two levels in reward probability and expected pallet.

	Level		Mean Diff.	Std. Error	*Z*-value	*P*-value
	Prob. (a)	Prob. (b)	(b) - (a)			
Reward Probability	0.25	0.50	0.0252	0.0250	1.01	0.312
	0.25	0.75	0.129	0.0143	9.03	***$P < 10^{-6}$
	0.50	0.75	0.104	0.0205	5.07	***$P < 10^{-6}$
	Exp. Pellet (a)	Exp. Pellet (b)	(b) - (a)			
Expected Pellet	0.25	0.50	0.00179	0.0314	0.0569	0.955
	0.25	0.75	-0.000556	0.0158	-0.0353	0.972
	0.25	1.50	0.0108	0.0223	0.483	0.629
	0.50	0.75	-0.00235	0.0272	-0.0862	0.931
	0.50	1.50	0.00898	0.0222	0.405	0.685
	0.75	1.50	0.0113	0.0158	0.718	0.473

Significance level is adjusted by Bonferroni correction. ***$P < 0.001$.

Supplementary Table 2 | Difference of means between two levels in reward delay condition.

	Level		Mean Diff.	Std. Error	Z-value	P-value
	Delay (a)	Delay (b)	(b) - (a)			
Reward delay condition	3	6	-0.0144	0.0193	-0.747	0.455
	3	10	-0.0269	0.0221	-1.22	0.223
	3	4-6-8	0.0528	0.0193	2.73	**0.00629
	3	2-6-10	0.208	0.0189	11.0	***$P < 10^{-6}$
	6	10	-0.0125	0.0228	-0.549	0.583
	6	4-6-8	0.0672	0.0201	3.35	***0.000807
	6	2-6-10	0.222	0.0196	11.3	***$P < 10^{-6}$
	10	4-6-8	0.0797	0.0228	3.50	***0.000464
	10	2-6-10	0.234	0.0223	10.5	***$P < 10^{-6}$
	4-6-8	2-6-10	0.155	0.0196	7.91	***$P < 10^{-6}$

Significance level is adjusted by Bonferroni correction. **$P < 0.01$, ***$P < 0.001$.
